# Cardiac‐specific overexpression of miR‐122 induces mitochondria‐dependent cardiomyocyte apoptosis and promotes heart failure by inhibiting Hand2

**DOI:** 10.1111/jcmm.16544

**Published:** 2021-05-04

**Authors:** Yajuan Shi, Zhi Zhang, Qiqi Yin, Chen Fu, Andrew Barszczyk, Xiaofu Zhang, Jiabing Wang, Deye Yang

**Affiliations:** ^1^ Division of Cardiology The Affiliated Hospital of Hangzhou Normal University Hangzhou China; ^2^ Division of Cardiology The First People’s Hospital of Yuhang District Hangzhou China; ^3^ Department of Internal Medicine The Third People's Hospital at Anji Huzhou China; ^4^ Department of Physiology University of Toronto Toronto ON Canada

**Keywords:** Hand2, heart failure, miR‐122, mitochondria‐dependent apoptosis, mitochondrial fission protein Drp1

## Abstract

MicroRNA‐122 (miR‐122) is one of several microRNAs elevated in heart failure patients. To investigate the potential role and mechanism of miR‐122 in heart failure, we constructed a transgenic mouse overexpressing miR‐122 in the heart. This mouse exhibited cardiac dysfunction (as assessed by transthoracic echocardiography), morphological abnormalities of the heart and cardiomyocyte apoptosis characteristic of heart failure. Mechanistically, we identified the Hand2 transcription factor as a direct target of miR‐122 using a dual‐luciferase reporter assay. In Tg‐miR‐122 mice and H9C2 cells with miR‐122 mimics, we detected apoptosis and increased expression of dynamin‐related protein‐1 (Drp1). This effect was blocked with prior knockdown of Hand2 in vitro. Our work suggests that miR‐122 causes cardiomyocyte apoptosis by inhibiting Hand2 and consequently increasing Drp1‐mediated mitochondrial fission. Such a mechanism likely contributes to heart failure and so modulating this pathway could be therapeutically valuable against heart failure.

## 1 INTRODUCTION

Heart failure is characterized by the functional loss of ventricular contractility. It occurs following hypertrophy and ventricle deterioration resulting from cardiomyocyte apoptosis.^1^ An early component of cardiomyocyte apoptosis in heart failure is the dysregulation of mitochondrial fission and fusion.^2^, ^3^, ^4^, ^5^, ^6^, ^7^ The relative rates of fusion and fission are normally tightly controlled, but during apoptosis, there is a dysregulation of key regulatory proteins like MFF, MFN1, MFN2, OPA1 and Drp1.^8^ Much work has focused on characterizing the regulatory pathways governing mitochondrial fission and fusion in terms of how they become dysregulated in heart failure and in terms of identifying molecular targets for potentially blocking this dysregulation.

MicroRNAs have gained significant attention as modulators of mitochondrial fission and fusion. MicroRNAs are endogenous, single‐stranded, short RNA sequences (~22 nucleotides) that regulate target gene expression by binding to the 3' untranslated regions (UTRs) of target mRNAs.^9^ The microRNAs miRNA‐499, miRNA‐140 and miRNA‐30c have been shown to modulate mitochondrial fission and affect cardiomyocyte apoptosis.^10^, ^11^, ^12^, ^13^ For example, miR‐499 inhibits cardiomyocyte apoptosis and reduces myocardial infarction size by inhibiting mitochondrial fission mediated by the Drp1‐mediated apoptotic pathway.^14^

MicroRNA‐122 (miR‐122) is also a particularly interesting microRNA that is conserved among vertebrates. Elevated levels of miR‐122 are associated with heart failure,^15^, ^16^, ^17^, ^18^ we well as acute myocardial infarction ^19^, ^20^ and acute coronary syndrome.^21^ We recently found that miR‐122 is up‐regulated in the Pax‐8 gene knock‐out mouse, which develops left ventricle enlargement and apoptosis ^22^ (similar to heart failure). On a cellular level, this early exosomal miRNA is associated with oxidative stress and mitochondria‐dependent apoptosis.^23^ However, it remains unknown whether miR‐122 also affects cardiac function via this apoptosis. Its mechanism of action also remains unknown, although sequence analysis suggests that miR‐122 interacts with the Hand2 transcription factor. Other miRNAs associated with heart failure also regulate Hand2.^24^ The Hand2 transcription factor normally plays an essential role in cardiac morphogenesis,^22^, ^25^, ^26^ with disruption or loss of Hand2 causing impaired cardiac development, apoptosis of cardiomyocytes and cardiac hypertrophy.^26^, ^27^, ^28^, ^29^ Further, mutations in the gene are prevalent in patients with congenital heart disease.^30^ We propose that Hand2 functions as an upstream regulator of mitochondrial fission.

The present study investigated whether miR‐122 induces cardiac dysfunction and morphological abnormalities (in addition to apoptosis), and whether its mechanism of action involves Hand2 and the Drp1‐mediated apoptotic pathway (apoptosis via excessive mitochondrial fission). We addressed the first question by overexpressing miR‐122 in a transgenic mouse model, primary cardiomyocytes and a cardiomyocyte cell line and then determining whether there were negative effects on heart function, heart and heart tissue morphology, and the presence of cellular apoptosis. We addressed the mechanistic question by assessing whether Hand2 mRNA or protein expression was reduced by miR‐122 overexpression in the mouse and in H9C2 cells. We further determined whether miR‐122 binds Hand2 directly using a dual‐luciferase reporter assay. Finally, we determined whether Drp1 mRNA or protein expression was increased in these models (suggesting involvement of the Drp1 apoptotic pathway), and whether any miR‐122 induced changes in Drp1 expression could be blocked by inhibiting Hand 2 (suggesting that Hand2 mediates the effect of mIR‐122 on Drp1).

## 2 METHODS

### 2.1 Tg‐miR‐122 transgenic mice

We created H11‐CAG‐LSL‐miR‐122‐PolyA knock‐in mice using the CRISPR/Cas9 system. We co‐injected Cas9 mRNA, small guiding RNA (5'‐CTGAGCCAACAGTGGTAGTA‐3'), and donor RNA into zygotes. The single guide RNA directed Cas9 endonuclease cleavage at the H11 locus to create a double‐strand break, and the repair of this break resulted in the insertion of CAG‐LSL‐Mir122‐PolyA at the H11 locus. We bred the resulting pups with α‐MHC Cre± mice to produce heart‐specific knock‐in mice. We ultimately obtained six genotypes of mice, miR‐122^fl/fl^/Cre^+^, miR‐122^fl/fl^/Cre^−^, miR‐122^fl/+^/Cre^+^, miR‐122^fl/+^/Cre^−^, miR‐122^+/+^/Cre^+^ and miR‐122^+/+^/Cre^−^ mice, of which miR‐122^fl/fl^/Cre^+^ and miR‐122^+/+^/Cre^−^ were used for the further experimental studies at eight weeks.

### 2.2 Transthoracic echocardiography

We performed two‐dimensional M‐mode echocardiography using a Vevo 770 high‐resolution in vivo imaging system with a 30‐MHz transducer on isoflurane‐anesthetized mice. The left ventricular (LV) end‐diastolic internal diameter (LVIDd), LV end‐systolic internal diameter (LVIDs), LV posterior wall thickness at the end of diastole (LVPWd) and LV anterior wall thickness at the end of diastole (LVPWs) were measured using M‐mode recordings. Left ventricular fractional shortening (FS) was calculated as FS percentage equals (LVIDd‐LVIDs)/LVIDd ×100%.

### 2.3 Morphological analysis

We treated mouse heart tissue with the paraffin embedding method, fixing with 4% paraformaldehyde before dehydration and embedding. The paraffin sections were stained with haematoxylin‐eosin (HE), and myocardial fragmentation area was measured with ImageJ software (Media Cybernetics INC.). The percentage of myocardial fragmentation was calculated as myocardial fragmentation area/total area ×100% in each field for at least three random fields.

### 2.4 Cell culture and transfection

We isolated primary cardiomyocytes from the hearts of neonatal C57/B6 mice and purchased H9C2 cells from the Cell Resource Center of the Shanghai Institute of Life Sciences, Chinese Academy of Sciences. We cultured both cell types in 90% Dulbecco's modified Eagle's medium (DMEM) (Gibco) with 10% foetal bovine serum (FBS) (Gibco) and 1% streptomycin (Thermo Fisher Scientific). Cells were maintained in an incubator with 95% air and 5% CO_2_ at 37°C. We transfected H9C2 cells with either NC‐miR‐122, miR‐122 mimics, NC‐OE, Hand2‐OE, si‐NC or si‐Hand2, followed by incubation for another 24 hours.

### 2.5 Protein analysis

Total protein was collected in RIPA buffer (Beyotime). Protein (25‐50 μg) was separated by electrophoresis on a 10% SDS polyacrylamide gel and then transferred to a nitrocellulose membrane. Membranes were then incubated at 4°C overnight with anti‐Hand2 (#ab56590, Abcam), anti‐Drp1 (#8570, CST), anti‐Bcl‐2 (#3498‐T, CST) or β‐actin (#4970, CST) primary antibodies, which were diluted to 1:1000 in TBST buffer. The membranes were then incubated with secondary antibodies (#A0208, Beyotime) for 1h. Images were scanned with a MicroChemi 4.2 (DNR) system. Western blot bands were quantified by measuring band intensity using ImageJ.

### 2.6 RNA analysis

Total RNA was prepared using the miRNA extraction kit (Tiangen). miR‐122 levels were measured through qRT‐PCR with the miRCute Enhanced Fluorescence Quantitative Assay Kit (Tiangen). U6 was employed as the loading control (forward: 5'‐GCTTCGGCAGCACATATACTAA‐3' and reverse: 5'‐AACGCTTCACGAATTTGCGT‐3'). cDNAs were synthesized using a cDNA synthesis kit (Takara) for the quantitative RT‐PCR assay. The primers used were as follows: Hand2: 5'‐CGCAGGACTCAGAGCATCAA‐3' and 5'‐TCACCAGCCTCGATCCCTTA‐3'; Drp1:5'‐CAGAATGAGAGGTCTCCGGG‐3' and 5'‐CTGCTTTGCTCGTGTAGTCTG‐3'; Bcl2:5'‐GTCGCTACCGTCGTGACTTC‐3' and 5'‐CAGACATGCACCTACCCAGC‐3'; GAPDH: 5'‐TGTGTCCGTCGTGGATCTGA‐3' and 5'‐CCTGCTTCACCACCTTCTTGA‐3'.

### 2.7 Dual‐luciferase reporter assay

The wild‐type 3'‐untranslated region (UTR) of Hand2 was amplified by PCR using genomic DNA of 293T cells, which contained the binding site of miR‐122. The corresponding mutant 3'‐UTR was produced by altering the seed region of the binding site of miR‐122. Then, the wild‐type and mutant 3 '‐UTR were respectively cloned into the psiCHECK‐2 luciferase vector. The miR‐122 mimic or miR‐122‐NC and luciferase plasmid containing wild‐type or mutant 3'‐UTR were co‐transfected into 293T cells. Luciferase activity was measured 48 hours after transfection using the dual‐luciferase reporter assay system (Promega).

### 2.8 TUNEL staining assay

We placed tissue sections on coverslips, washed them twice in paraxylene and rinsed them with a graded series of ethanol. Then, we incubated them in 21%‐37% proteinase K solution for 15‐30 minutes. We then washed them in phosphate‐buffered saline (PBS) and incubated them for 60 minutes at 37℃ in a dark humidified environment with or without 50µL of TUNEL reaction mixture. We added DAPI after sections were fully converted with POD. We then washed the sections in PBS again and immediately visualized and quantified TUNEL‐positive cells using a fluorescence microscope.

### 2.9 Measurement of mitochondrial membrane potential

We measured mitochondrial membrane potential (MMP) using the JC‐1 Kit (Beyotime) following the manufacturer's protocol. We collected the H9C2 cells, washed them with PBS and then resuspended them in DMEM. We added 0.5 mL JC‐1 staining solution and incubated the cells at 37℃ for 20 minutes. We then precipitated and washed the cells twice with JC‐1 buffer before resuspending the cells in JC‐1 staining buffer. We assessed the colour of the JC‐1 dye using flow cytometry, with red and green fluorescence indicating high and low MMP, respectively.

### 2.10 Annexin V‐FITC/PI double staining assay

We determined the proportion of apoptotic cells using the Annexin V‐FITC Apoptosis Kit (Beyotime) according to the manufacturer's protocol. After transfecting cells for 36 hours, we cultured them in DMEM for 12 hours. We then harvested and washed the cells with PBS buffer and resuspended them in the kit's staining buffer. We added 5µL of Annexin V‐FITC and 10 µL of propidium iodide (PI) to the cells at room temperature for 5 minutes. We analysed the mixture of cells using FACScan flow cytometry (BD Biosciences) to quantify the proportion of apoptotic (Annexin V+ and PI‐) cells.

### 2.11 Immunofluorescence staining

We rinsed the cells three times using 1×PBS, fixed them with 4% paraformaldehyde for 20 minutes at room temperature and then permeabilized them with 0.1% Triton X‐100 in PBS for 20 minutes. Blocking was performed with 5% BSA for 30 minutes at room temperature, and cells were incubated with DRP1 (CST) at 4°C overnight. The next day, we incubated the cells with Alexa Fluor donkey‐anti‐rabbit IgG secondary antibody (Thermo Fisher) for 2 hours at 4°C in the dark. We then counterstained with DAPI to visualize nuclei. Finally, we visualized fluorescence using an LSM 880 model confocal microscope (Carl Zeiss).

## 3 RESULTS

### 3.1 Cardiac‐specific overexpression of miR‐122 induces cardiac dysfunction consistent with heart failure in transgenic mice

**FIGURE 1:**
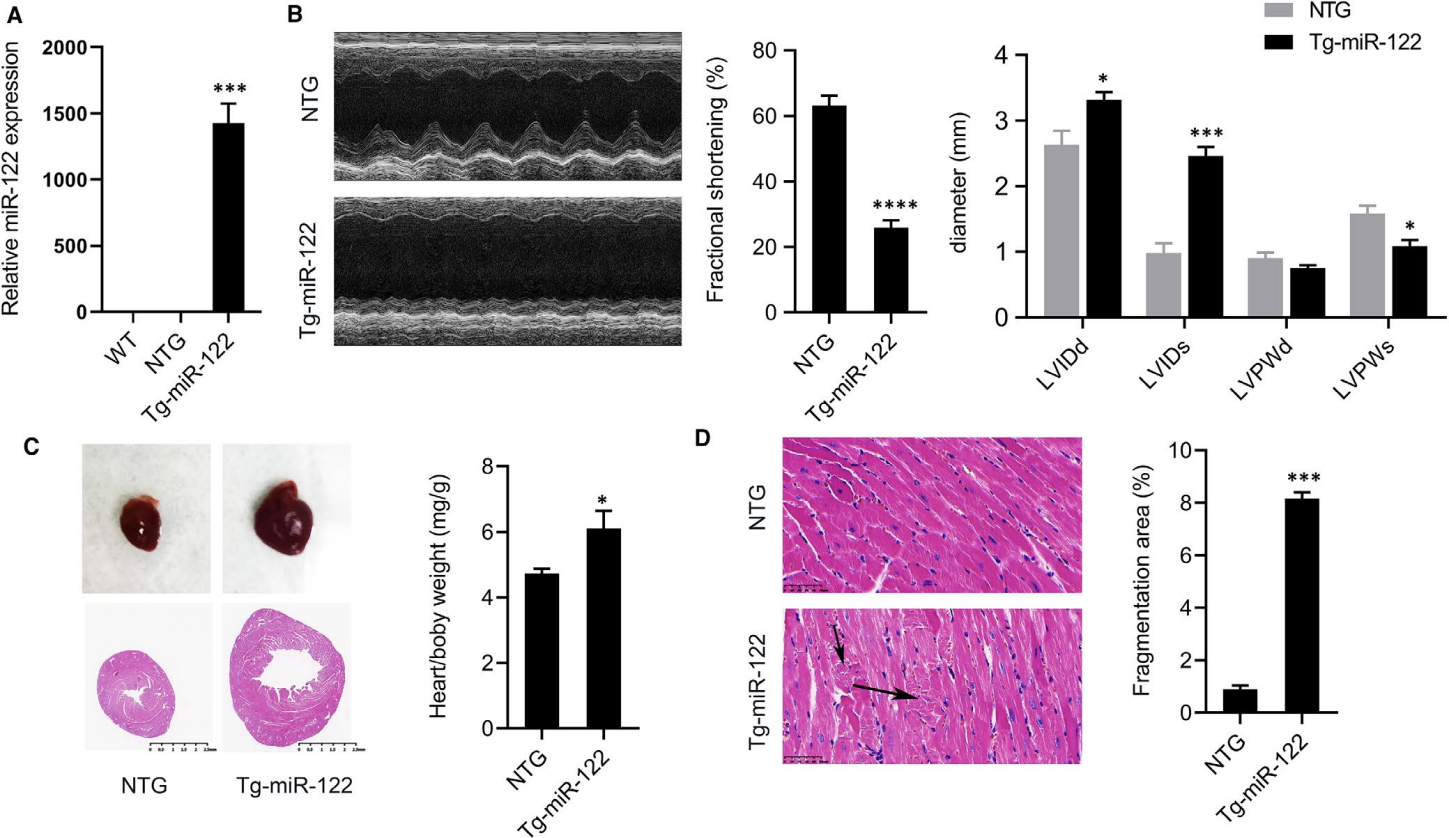
Cardiac overexpression of miR‐122 induced functional deficits consistent with heart failure. (A) Relative expression of miR‐122 in the hearts of Tg‐miR‐122 mice, NTG and wild‐type mice. (B) Representative M‐mode images of Tg‐miR‐122 mice and NTG mice. We quantified the ratio of left ventricular fractional shortening (FS), left ventricular internal diameters at diastole (LVIDd) and systole (LVIDs), and left ventricular wall thickness at diastole (LVPWd) and systole (LVPWs). (C) Haematoxylin/eosin (HE) staining of heart tissue. Scale bar: 2.5mm. Ratios of heart weight to bodyweight (HW/BW) in Tg‐miR‐122 and NTG mice. (D) Representative images of haematoxylin/eosin‐stained heart tissues from Tg‐miR‐122 and NTG mice. Scale bar: 50 μm. N = 5 samples per group. *P < 0.05, ***P < 0.005 for all panels vs NTG mice. An unpaired t test was used to assess significance. Data are means ± SEM

Since past studies showed increased miR‐122 expression is increased in heart disease, we set out to study the role of miR‐122 in the heart by creating a transgenic mouse with cardiac overexpression of miR‐122. Our Tg‐miR‐122 mice (miR‐122^fl/fl^/Cre^+^) indeed had increased expression of miR‐122 compared with the non‐transgenic (NTG) control mice (miR‐122^+/+^/Cre^‐^) (Figure 1A). We also measured the expression of miR‐122 in C57/B6 mouse hearts and found no difference between WT and NTG mice (Figure 1A). We characterized cardiac function in Tg‐miR‐122 and NTG mice using echocardiography (Figure 1B). We found that Tg‐miR‐122 mice had a statistically significant reduction in left ventricular fractional shortening (from 63% to 26%; *P* < 0.05) and a statistically significant increase in LVIDd (from 2.6mm to 3.4mm; *P* < 0.05) and LVIDs (from 1.0mm to 2.5mm; *P* < 0.05). LVPWd did not differ between Tg‐miR‐122 and NTG mice. Morphologically, Tg‐miR‐122 mice had visibly larger hearts and enlarged cardiac chambers (via HE staining) (Figure 1C) compared with NTG mice. Further, Tg‐miR‐122 had greater heart/bodyweight ratios compared with NTG mice (Figure 1C). As shown in Figure 1D, the myocardial morphology of Tg‐miR‐122 mice was altered, with evidence of myocardial rupture. Taken together, these data demonstrate that cardiac overexpression of miR‐122 impairs cardiac function and induces functional deficits consistent with heart failure in vivo.

### 3.2 miR‐122 overexpression induces apoptosis in vitro and in vivo

**FIGURE 2:**
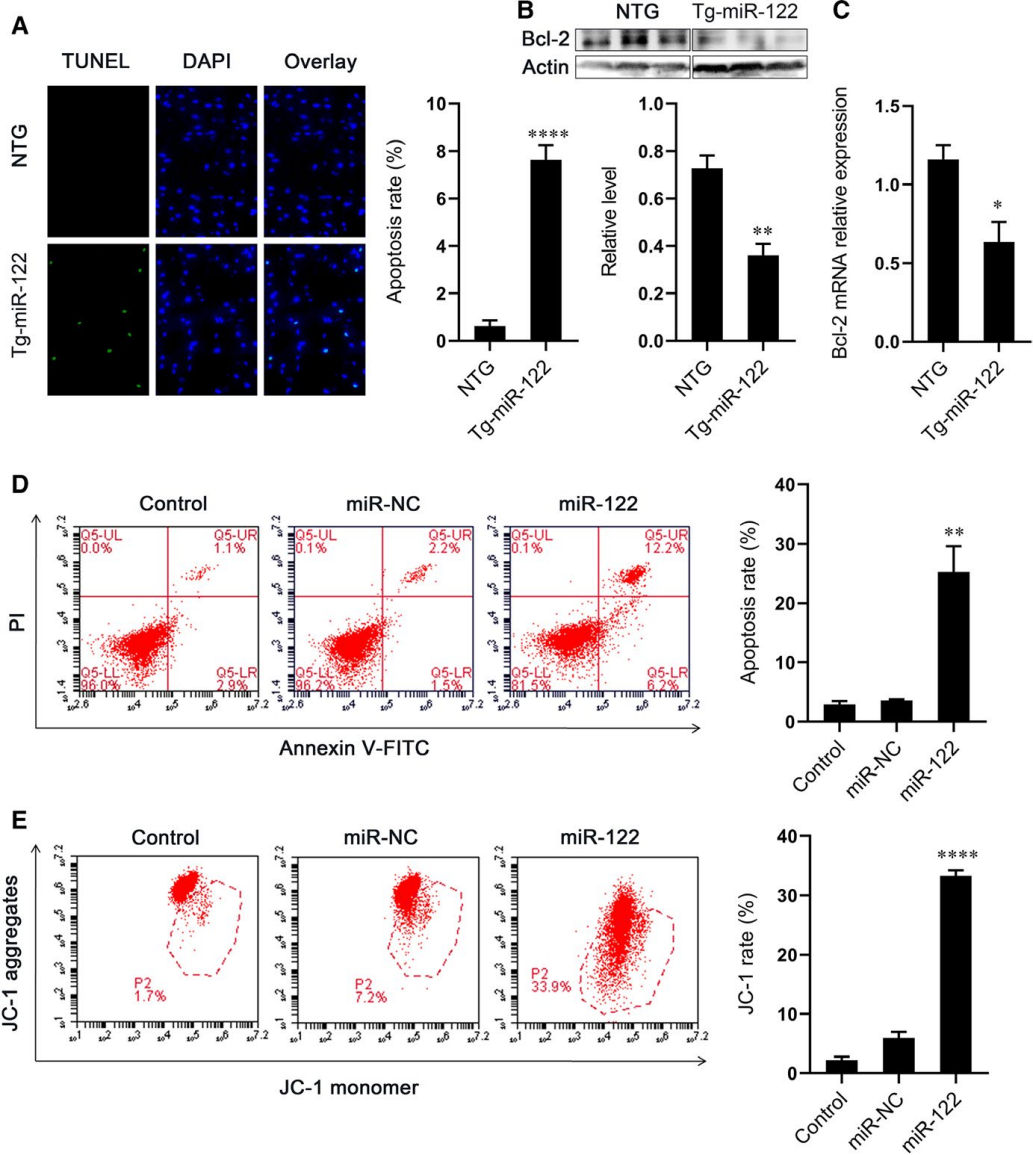
Overexpression of miR‐122 induced apoptosis of cardiomyocytes. (A) Left, representative cardiac sections from NTG and Tg‐miR‐122 mice. Green, TUNEL‐positive myocyte‐nuclei, blue, DAPI‐stained nuclei. Scale bar: 50 μm; right, quantitative analysis of apoptosis. (B) Protein expression of Bcl‐2 in the heart of NTG and Tg‐miR‐122 mice. Top, examples of Western blot bands; bottom, quantitative analysis. (C) mRNA expression of Bcl‐2. (D) Apoptosis in H9C2 cells. Left, representative images of Annexin V‐FITC/PI staining FACS assay. Cells in the lower right quadrant represent early apoptosis and those in the upper right quadrant represent late apoptosis; right, quantitative analysis of cellular apoptosis. (E) Effect of miR‐122 on mitochondrial membrane potential in H9C2 cells. Left, H9C2 cells were gated by flow cytometry with JC‐1. Red fluorescence indicates higher membrane potential; right, quantitative analysis of green/red ratio. N = 5 samples per group. *P < 0.05, **P < 0.01, ****P < 0.001 for all panels vs NTG mice or miR‐NC. An unpaired t test was used to determine statistical significance. Data are means ± SEM

Next, we investigated the effect of miR‐122 overexpression on apoptosis. We found that the hearts of Tg‐miR‐122 mice had significantly more apoptotic myocardial cells than NTG mice as indicated by the proportion of TUNEL‐positive cells (Figure 2A). Further, we found that the protein and mRNA expression of anti‐apoptotic factor Bcl‐2 was decreased in Tg‐miR‐122 mice (Figure 2B,C). H9C2 cells transfected with miR‐122 mimics also had more apoptotic cells compared to control cultures (Figure 2D). However, we found that miR‐122 mimic caused a green‐ward shift in JC‐1 immunofluorescence (Figure 2E), suggesting a reduction of mitochondrial membrane potential (such dysfunction is an early sign of apoptosis). These results demonstrate that miR‐122 overexpression causes the apoptosis of cardiomyocytes, which could potentially explain the impaired cardiac function in Tg‐miR‐122 mice.

### 3.3 miR‐122 overexpression regulates mitochondrial fission protein Drp1

**FIGURE 3:**
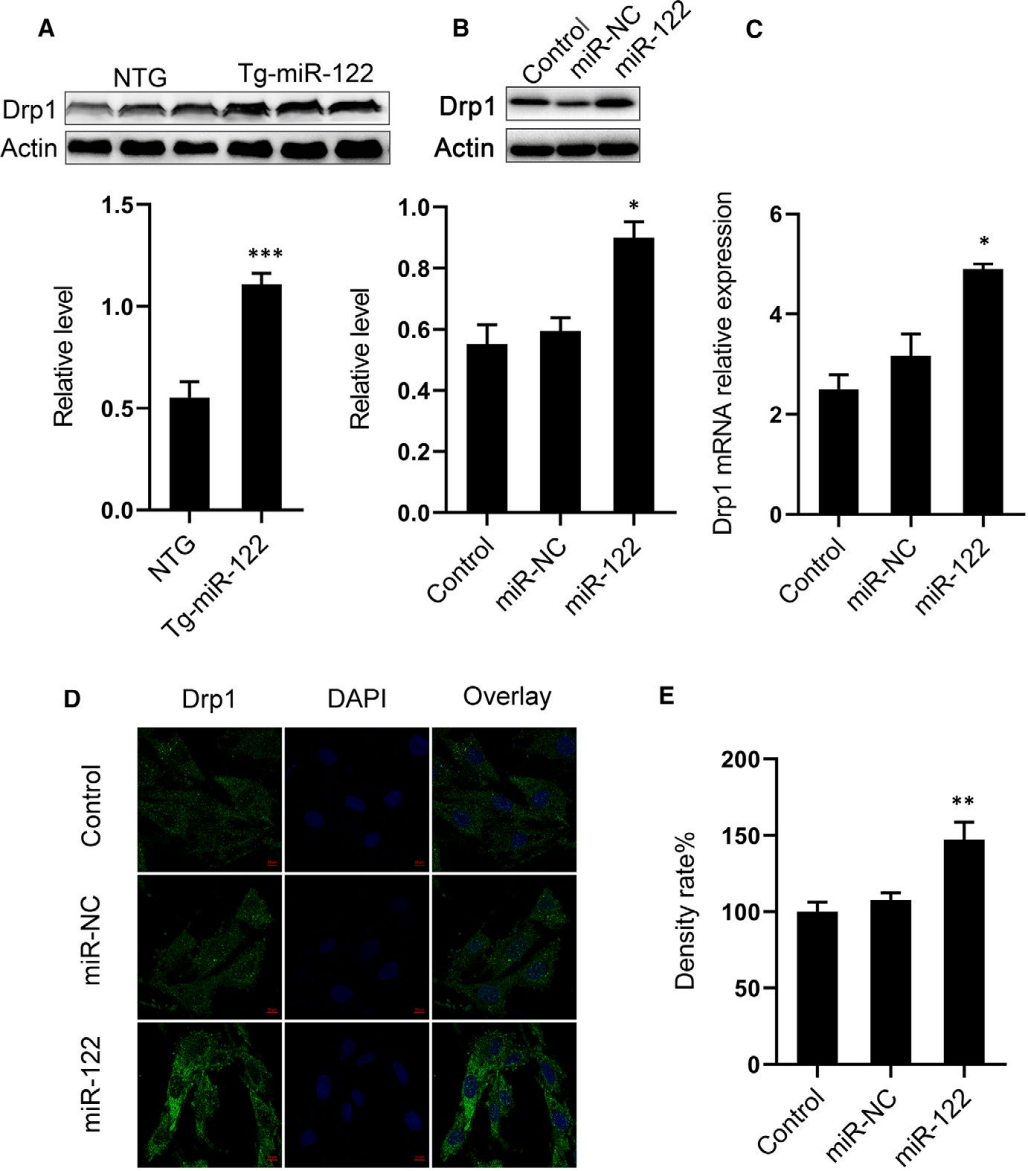
Overexpression of miR‐122 increased protein expression of Drp1. (A) Protein expression of Drp1 in the hearts of NTG and Tg‐miR‐122 mice. Top, Western blot example bands; bottom, quantitative analysis. (B) Protein expression of Drp1 in H9C2 cells transfected with miR‐122 mimics or miR‐NC. Top, examples of Western blot bands; bottom, densitometric analysis of protein level. (C) mRNA expression of Drp1 in H9C2 cells. (D) Representative images of H9C2 cells observed with a laser confocal microscope and cell sections treated with immunofluorescent staining. Scale bar: 50 μm. (E) Quantitative analysis of fluorescence. N = 5 samples per group. *P < 0.05, **P < 0.01, ***P < 0.005 for all panels vs NTG or miR‐NC. An unpaired t test was used to determine statistical significance. Data are means ± SEM

A growing body of evidence suggests that certain microRNAs cause apoptosis by abnormally regulating mitochondrial fission,^13^, ^14^, ^15^, ^16^ and so we investigated whether miR‐122 induces cardiomyocyte apoptosis by regulating mitochondrial fission protein Drp1. We quantified Drp1 protein expression in Tg‐miR‐122 and NTG mice via Western blot. Drp1 protein expression was significantly greater in Tg‐miR‐122 mice compared with NTG mice (Figure 3A). Moreover, we found that Drp1 protein and mRNA levels were elevated in H9C2 cells transfected with miR‐122 mimics compared with control culture (Figure 3B,C). The difference in protein levels was also evident via immunofluorescence and histochemical staining (Figure 3D,E). These results strongly suggest that miR‐122 overexpression induces apoptosis by regulating mitochondrial fission.

### 3.4 Hand2 is a direct target of miR‐122

**FIGURE 4:**
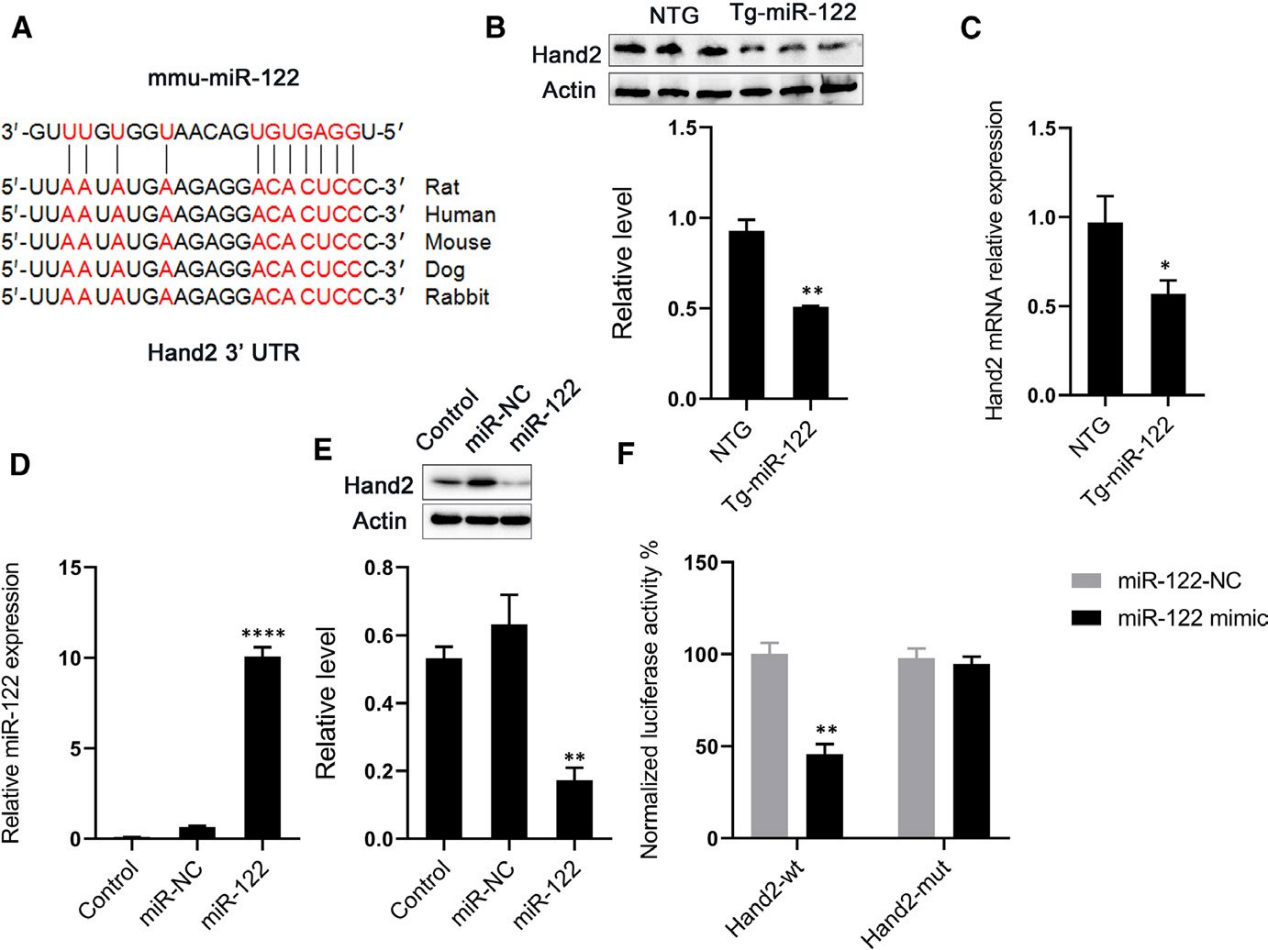
miR‐122 directly targets Hand2 to decrease Hand2 expression. (A) Putative binding sites for miR‐122 in the 3’‐UTR of Hand2 identified via bioinformatics program TargetScan. (B) Effect of miR‐122 on the protein expression of Hand2 in mice. Top, examples of Western blot bands; bottom, quantitative analysis. (C) mRNA expression of Hand2 in mice. (D) Expression of miR‐122 in H9C2 cells infected with miR‐NC or miR‐122 mimics. (E) Effect of miR‐122 mimics on the expression of Hand2 in H9C2 cells. Top, examples of Western blot bands; bottom, quantitation analysis. (F) Normalized luciferase ratios in 293T cells that were infected with miR‐NC or miR‐122 mimics, followed by transfection with plasmid constructs containing wild‐type (wt), mutation (mut) of the 3’‐UTR of Hand2. N = 5 samples per group. *P < 0.05, **P < 0.01, ****P < 0.0001 for all panels vs NTG or miR‐NC. An unpaired t test was used to determine statistical significance. Data are means ± SEM

To explore the mechanisms by which miR‐122 regulates apoptosis, we identified potential miR‐122 targets using the bioinformatics program TargetScan. We found that Hand2 had conserved binding sites for miR‐122 (Figure 4A). Hence, we hypothesized that Hand2 might also be a downstream target of miR‐122 that controls apoptosis. We found that the protein and mRNA expression of Hand2 significantly decreased in Tg‐miR‐122 mice (Figure 4B,C). We confirmed this by also investigating whether miR‐122 could influence the expression of Hand2 in H9C2 cells (Figure 4D). We found that miR‐122 mimics reduced protein expression of Hand2 in H9C2 cells compared with control transfection (Figure 4E). Next, we investigated whether Hand2 was a direct target of miR‐122 by measuring the effect of miR‐122 on the translation of Hand2 using a double luciferin reporter gene. miR‐122 mimics reduced the luciferase activity of the reporter gene of Hand2, whereas these reductions disappeared when the sequences of the 3'‐UTR binding site were mutated (Figure 4F). These results demonstrate that Hand2 is a direct target of miR‐122; such an interaction could potentially mediate the effect of miR‐122 on cardiomyocyte apoptosis.

### 3.5 Hand2 regulates Drp1 and apoptosis

**FIGURE 5:**
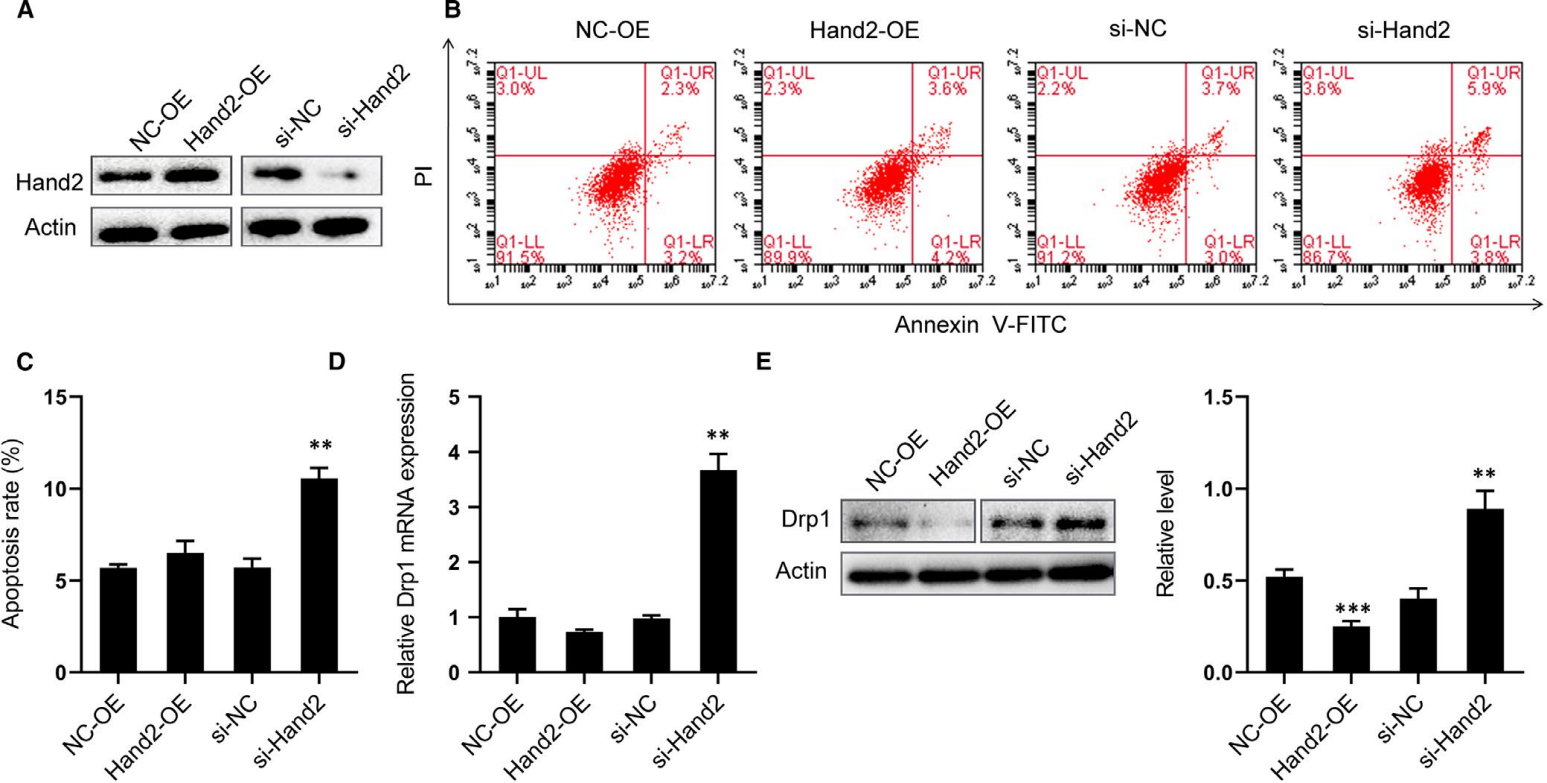
Hand2 silencing increases Drp1 expression and apoptosis demonstrated with H9C2 cells transfected with NC‐OE, Hand2‐OE, si‐NC or si‐Hand2. (A) Verification of transfection effect with Western blot. (B) Representative images of Annexin V‐FITC/PI staining FACS assay for apoptosis. Cells in the lower right quadrant represent early apoptosis and those in the upper right quadrant late apoptosis. (C) Quantitative analysis of apoptosis. (D) Representative expression of Hand2 mRNA in all groups. (E) Effect of Hand2 on the expression of Drp1 in H9C2 cells. Top, examples of Western blot bands; bottom, quantitation analysis. N = 5 samples per group. **P < 0.01, ***P < 0.005 for all panels vs NC‐OE or si‐NC. An unpaired t test was used to determine statistical significance. Data are means ± SEM

We have independently demonstrated that overexpression of miR‐122 inhibits the expression of Hand2 and also triggers Drp1‐mediated mitochondria‐dependent apoptosis. We will now investigate whether Hand2 mediates apoptosis via Drp1 by reducing Hand2 expression with siHand2 transfection or overexpressing Hand2 with Hand2‐OE plasmids in H9C2 cells (Figure 5A). We found that apoptosis increased significantly after Hand2 silencing, though it was unaffected by Hand2 overexpression (Figure 5B,C). Further, Western blotting and qRT‐PCR revealed that Drp1 protein and mRNA levels respectively were increased with siHand2 transfection. In contrast, overexpression of Hand2 decreased Drp1 protein expression but did not have an effect on mRNA levels (Figure 5D,E). The above results indicate that Hand2 could affect apoptosis by regulating Drp1 expression.

## 4 DISCUSSION

We found that overexpression of miR‐122 in vivo indeed induces functional deficits and morphological abnormalities resembling those in heart failure. We also found that overexpression of miR‐122 leads to apoptosis of cardiac cells both in vivo and in vitro. Together, these findings suggest that miR‐122 plays a causal role in cardiac dysfunction through the induction of cardiomyocyte apoptosis. Mechanistically, we found that overexpressing of miR‐122 increases expression of the Drp1 mitochondrial fission protein, suggesting that apoptosis (and cardiac dysfunction) occurs via Drp1‐mediated up‐regulation of mitochondrial fission. We further found that miR‐122 directly interacts with the miR‐122 transcription factor and that Hand2 mediates the effect of miR‐122 on Drp1. Together, this suggests that miR‐122 triggers apoptosis by inhibiting Hand2, which in turn causes an increase in the expression of Drp1, and ultimately, this results in too much mitochondrial fission and apoptosis. Thus, modulation of miR‐122 and Hand2 could potentially be therapeutically useful against heart failure or other diseases where miR‐122 plays a causal role.

It is well established that miR‐122 levels are elevated in heart failure,^15^, ^16^, ^17^, ^18^ but its specific role in cardiac function has not been characterized. While miR‐122 was previously known as a pro‐apoptotic factor in cardiomyocytes, we show here for the first time in vivo that miR‐122 causes cardiac dysfunction, morphological changes and apoptosis in rodents that strongly resemble the human heart failure phenotype. Thus, it is likely that miR‐122 is also a causal factor in the pathophysiology of human heart disease.

Further, the mechanism of the miR‐122 in apoptosis was not well understood. Here for the first time, we suggest a likely mechanism for miR‐122 in the form of increasing expression of the Drp1 mitochondrial fission protein. It is well known that cardiomyocyte damage (eg via hypoxia and reoxygenation) stimulates the up‐regulation of Drp1 and its translocation from cytoplasm to mitochondria^31^, ^32^, ^33^ to increase mitochondrial fission. This causes apoptosis^34^, ^35^ that is prevented by inhibiting Drp1.^7^ Our work here suggests that miR‐122 could be an upstream trigger of this Drp1 effect, with miR‐122 expression causing an increase in Drp1 expression. This mechanism is consistent with work on other microRNAs like miR‐499^14^ and miR‐23a,^36^ where such miRNAs trigger apoptosis of cardiomyocytes by up‐regulating Drp1‐meditated mitochondrial fission.

Our work goes a step further by identifying Hand2 as a mediator of this effect. We demonstrated that the Hand2 transcription factor is indeed a direct target gene of miR‐122, as we previously suggested.^22^ Although the Hand 2 transcription factor is a widely known regulator of cardiac development,^37^ studies have also shown that Hand2 plays a role in heart disease in postnatal mice.^38^ Our work here is the first to implicate Hand2 in rodent phenotypes resembling heart failure. Importantly, we found that Hand2 mediates the effect on Drp1. This pathway may be regulated upstream by calcineurin. This comes from evidence that protein expression of Hand2 is increased in transgenic mice expressing a mutant activated form of calcineurin^24^ and Drp1 is known to be regulated by the calcineurin catalytic subunit (CnA).^39^, ^40^, ^41^ Increased calcineurin signalling also results in mitochondrial dysfunction and heart failure.^42^ Further investigation is needed to elucidate the roles of each protein in the maintenance of mitochondrial homeostasis.

A limitation of our study was that while we identified the link between miR‐122 and a rodent phenotype resembling heart failure, we have not yet gone a step further to investigate whether the down‐regulation of miR‐122 has a protective effect. Second, although we identified Hand2 as a direct target of miR‐122, we have yet to determine how Hand2 regulates Drp1 function. Further investigation will be required to fully understand the Hand2/Drp1 pathway.

Although the impact of miR‐122 overexpression on cardiomyocyte apoptosis is clear, future studies should assess right ventricular function (in addition to left ventricular function) to provide a more comprehensive picture of cardiac dysfunction. Future studies should also aim to address the remaining questions about this mechanism and how it affects mitochondrial dynamics. Given that our findings suggest cardiomyocyte apoptosis (as a result of miR‐122 overexpression) occurs through the Drp1‐mediated pathway, future studies should investigate the mechanisms by which Drp1 is activated. One known trigger of Drp1‐mediated mitochondrial fission is high levels of reactive oxygen species (ROS), while low levels of ROS have been found to promote mitochondrial fusion.^43^ High levels of ROS are present in heart failure^44^ and are thought to contribute to the pathogenesis of heart disease.^45^ Therefore, future studies should also investigate how levels of ROS affect this pathway. Finally, future studies of this pathway could employ wheat germ agglutinin (WGA) staining to quantify fibrotic damage in cardiomyocytes, and additional insights might be gained by evaluating the presence of hypertrophy at the cellular level (eg in H9C2 cells) in addition to the tissue level.

In summary, we found that the miR‐122 induces functional deficits and morphological abnormalities resembling heart failure in mice and that it induces cardiomyocyte apoptosis. Our work suggests that this occurs via a Hand2/Drp1 mitochondrial fission pathway and thus provides an understanding of how miR‐122 affects cardiac function. Such a mechanism likely contributes to heart failure. Future work should investigate whether modulating this pathway is therapeutically valuable against heart failure.

## CONFLICTS OF INTEREST

The authors declare no conflicts of interest.

## AUTHOR CONTRIBUTIONS

**Yajuan Shi:** Data curation (equal); Resources (equal); Writing‐original draft (equal). **Zhi Zhang:** Data curation (equal); Writing‐original draft (equal). **Qiqi Yin:** Data curation (equal); Writing‐review & editing (equal). **Chen Fu:** Formal analysis (equal); Validation (equal). **Andrew Barszczyk:** Methodology (equal); Writing‐review & editing (equal). **Xiaofu Zhang:** Methodology (equal); Software (equal). **Jiabing Wang:** Data curation (equal); Software (equal). **Deye Yang:** Project administration (equal); Supervision (equal); Writing‐review & editing (equal).

## Data Availability

The data that support the findings of this study are available from the corresponding author upon reasonable request.
